# 4-Hydroxynonenal Modulates Blood–Brain Barrier Permeability In Vitro through Changes in Lipid Composition and Oxidative Status of Endothelial Cells and Astrocytes

**DOI:** 10.3390/ijms232214373

**Published:** 2022-11-19

**Authors:** Marina Cindrić, Ana Čipak Gašparović, Lidija Milković, Ivana Tartaro Bujak, Branka Mihaljević, Neven Žarković, Kamelija Žarković

**Affiliations:** 1 Laboratory for Oxidative Stress, Division of Molecular Medicine, Rudjer Boskovic Institute, Bijenička 54, 10000 Zagreb, Croatia; 2 Division of Pathology, School of Medicine, University of Zagreb, Šalata 10, 10000 Zagreb, Croatia; 3 Division of Pathology and Cytology, University Hospital Centre Zagreb, Kišpaticeva 12, 10000 Zagreb, Croatia; 4 Radiation Chemistry and Dosimetry Laboratory, Division of Materials Chemistry, Rudjer Boskovic Institute, Bijenička 54, 10000 Zagreb, Croatia

**Keywords:** blood–brain barrier, astrocytes, 4-hydroxynonenal, lipid composition

## Abstract

Blood brain barrier (BBB) is a dynamic interface responsible for proper functioning of brain, but also a major obstacle for effective treatment of neurological diseases. Increased levels of free radicals, in high ferrous and high lipid content surrounding, induce lipid peroxidation, leading to production of 4-hydroxynonenal (HNE). HNE modifies all key proteins responsible for proper brain functioning thus playing a major role in the onset of neurological diseases. To investigate HNE effects on BBB permeability, we developed two in vitro BBB models–‘physiological’ and ‘pathological’. The latter mimicked HNE modified extracellular matrix under oxidative stress conditions in brain pathologies. We showed that exogenous HNE induce activation of antioxidative defense systems by increasing catalase activity and glutathione content as well as reducing lipid peroxide levels in endothelial cells and astrocytes of ‘physiological’ model. While in ‘pathological’ model, exogenous HNE further increased lipid peroxidation levels of endothelial cells and astrocytes, followed by increase in Nrf2 and glutathione levels in endothelial cells. At lipid composition level, HNE caused increase in ω3 polyunsaturated fatty acid (PUFA) level in endothelial cells, followed by decrease in ω3 PUFA level and increase in monounsaturated fatty acid level in astrocytes. Using these models, we showed for the first time that HNE in ‘pathological’ model can reduce BBB permeability.

## 1. Introduction

The blood–brain barrier (BBB) is a sophisticated system of brain capillaries, their basal membrane and surrounding cells. Working together, these parts make the neurovascular unit responsible for the physical, metabolic and transport properties of the barrier [1]. Although considered a dynamic interface responsible for the proper functioning of the central nervous system (CNS) [2], BBB is frequently looked at as a major obstacle to the effective treatment of neurological diseases [3]. Since a majority of the literature describes impaired integrity of the BBB under most CNS pathologies [4,5], the question arises how come the treatment of these diseases is not facilitated then. One of the possible explanations is that almost every CNS disease is revealed at a later stage when treatment is less effective due to the onset of the disease. Additionally, there is a possibility that at an early stage of the disease, BBB integrity is not altered or is even strengthened. For example, in the case of CNS tumors, BBB permeability increases only at higher tumor grades (i.e., grades III and IV) [6]. Besides, even though compromised integrity can be transient, very often, due to the severity of brain disorders, it can become permanent [7]. The brain then becomes a non-privileged site that has lost internal control and needs to face direct contact with the rest of the organism through the blood. This leads to oxidative stress increase and pronounced damage to the major cell systems in case of inadequate antioxidative protection. Oxidative stress plays a major role in the regulation of BBB permeability through affecting tight junctions (TJ) and the extracellular matrix (ECM) [8,9]. Furthermore, the brain is susceptible to oxidative stress due to its high polyunsaturated fatty acid (PUFA) content [10]; therefore, changes in fatty acid composition or their oxidative status can influence the arrangement of TJ proteins and consequently BBB integrity [11,12]. Indeed, lipid hydroperoxides and reactive aldehydes that arise from the lipid peroxidation of PUFAs in cell membranes can induce transient or permanent changes of membrane or ECM constituents, thus additionally influencing the BBB integrity [13]. One of the most explored reactive aldehydes, 4-hydroxynonenal (HNE), was shown to play a major role in the onset of neurodegenerative diseases [14,15], but also in CNS tumors [16,17]. Proteins modified by HNE in these diseases could be categorized into several groups, including cytoskeletal integrity, mitochondrial dysfunction, stress response, antioxidant defense, energy metabolism, protein synthesis, fatty acid transport and neuronal communication [15,18]. The presence of HNE-modified proteins was described not only in brain cells but also in the stroma, especially in CNS tumors, where HNE presence positively correlates with tumor grade in the vascular wall as well as in the stroma [16]. Effects of HNE on vascular endothelial and smooth muscle cells range from beneficial, through adaptive to pathological [19]. At low physiological levels, HNE exerts positive effects on cell function through targeting specific cellular pathways. Higher HNE concentrations will induce an adaptive response of vascular cells through activation of the major antioxidative transcription factor, Nrf2, in order to minimize or prevent loss of cell function. Finally, at pathological concentrations, HNE induces pro-inflammatory and pro-apoptotic signals [19].

Taking together the above described and the fact that ECM is equally important for the maintenance of BBB integrity as are TJs [20], both being affected by HNE in CNS pathologies, our aim was to develop two in vitro BBB models (‘physiological’ and ‘pathological’). The ‘physiological’ model was developed as a co-culture of rat astrocytes and rat brain endothelial cells on collagen-coated cell culture inserts. The ‘pathological’ model was developed on collagen-coated inserts treated with 10 µM HNE to mimic modified ECM under oxidative stress conditions in CNS pathologies. Using these two models, we have shown for the first time that HNE in the ‘pathological’ model may reduce BBB permeability through changes in the oxidative status and PUFA composition of both astrocytes and endothelial cells. Results shown here could give new insights into possible explanations for treatment failure of CNS diseases.

## 2. Results

### 2.1. Effects of HNE on the Viability, Proliferation and Oxidative Status of RbE4 Cells

**Figure 1:**
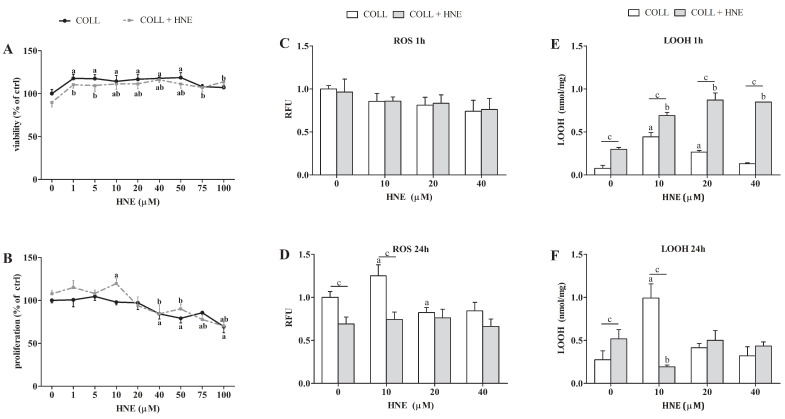
Sensitivity of RbE4 cells on HNE treatment on collagen (COLL) and collagen treated with 10 µM HNE (COLL + HNE). (**A**) Viability of RbE4 cells assessed by MTT assay after HNE treatment (0, 1, 5, 10, 20, 40, 50, 75, 100 µM) and represented as % of control. (**B**) Proliferation of RbE4 cells measured by 3H thymidine assay after HNE treatment (0, 1, 5, 10, 20, 40, 50, 75, 100 µM) and represented as % of control. ROS levels (**C**) 1 h and (**D**) 24 h after HNE treatment (0, 10, 20, 40 µM) represented in RFU (relative fluorescence unit). LOOH measured 1 h (**E**) and 24 h (**F**) after HNE treatment (0, 10, 20, 40 µM). All results are represented as mean ± SD, *n* = 12 samples in each group from 3 independent experiments. a—statistically significant in respect to control (0) on pure collagen, *p* < 0.001; b—statistically significant in respect to control (0) on modified collagen, *p* < 0.001; c—statistically significant between groups of same treatment, *p* < 0.001.

The sensitivity of RbE4 cells to a wide range of HNE concentrations was determined by viability and proliferation assays in order to select appropriate concentrations for further analyses. While HNE concentrations below 10 µM had no effect on cell proliferation and a significant decrease in cell proliferation occurred at 40 µM HNE (Figure 1B), these two concentrations, together with 20 µM HNE, were selected for the induction of oxidative stress in RbE4 cells. Therefore, the first series of experiments was performed with RbE4 cells grown on native collagen to obtain the lowest HNE concentration that would increase oxidative stress, but without inducing cell death. This was achieved with 10 µM HNE (Figure 1D–F), which was thus selected for collagen modification.

Effects of HNE on viability and proliferation of RbE4 cells grown on native collagen and collagen modified with 10 µM HNE in parallel were determined by measuring metabolic activity (Figure 1A) and incorporation of 3H-tymidine (Figure 1B). HNE concentrations ranging from 1 to 50 µM increased the viability of the RbE4 cells grown on both surfaces (*p* < 0.001). The proliferation assay results did not correlate with the MTT results. An increase in proliferation of RbE4 cells grown on HNE-modified collagen was observed at 10 µM HNE (*p* < 0.001), while from 40 to 100 µM HNE decreased RbE4 proliferation on both surfaces.

The three selected HNE concentrations showed different effects on the proliferation of RbE4 cells grown on modified collagen, ranging from stimulation (10 µM) to no effect (20 µM) and to decrease in proliferation (40 µM).

In order to determine the effect of selected HNE concentrations on the oxidative status of RbE4 cells on HNE-modified collagen in parallel with native collagen and prior to experiments on BBB models, reactive oxygen species (ROS) (Figure 1C,D) and lipid hydroperoxides (LOOH) measurements were performed (Figure 1E,F) 1 h and 24 h after HNE treatment. One hour after HNE treatment, there were no changes in ROS levels, regardless of surface or treatment. Significant changes in ROS levels occurred only in RbE4 cells grown on collagen 24 h after HNE treatment. Interestingly, 10 µM HNE increased ROS levels (*p* < 0.001), while 20 µM HNE decreased ROS levels (*p* < 0.001). In RbE4 cells grown on HNE-modified collagen, ROS levels remained constant regardless of treatment. LOOH were also measured at 1 h and 24 h after HNE treatment on cells grown on both surfaces (Figure 1E,F). One hour after HNE treatment, RbE4 cells grown on HNE-modified collagen had higher LOOH concentrations than cells grown on native collagen (*p* < 0.001). Unlike the ROS and LOOH levels 1 h after treatment, RbE4 cells grown on native collagen after 24 h had an increase in LOOH concentrations caused by 10 µM and 20 µM HNE (*p* < 0.001), while 40 µM HNE had no effect on LOOH formation. In RbE4 cells grown on HNE-modified collagen, the addition of HNE caused a concentration-dependent LOOH increase (*p* < 0.01). Further, 24 h exposure to HNE of RbE4 cells grown on native collagen showed similar trends in LOOH concentrations, as did ROS. The highest increase in LOOH concentrations (*p* < 0.001) was observed in RbE4 cells on native collagen treated with 10 µM HNE, while the same HNE concentration decreased LOOH concentrations in RbE4 cells grown on HNE-modified collagen (*p* < 0.001).

### 2.2. Sensitivity of RbE4 Cells and Astrocytes in Two BBB Models to Selected HNE Concentrations

**Figure 2:**
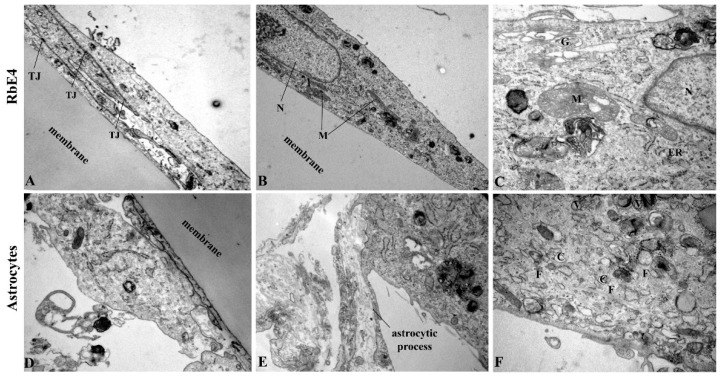
TEM pictures of RbE4 cells and astrocytes grown on cell culture inserts. (**A**–**C**) Endothelial RbE4 cells show characteristic oval shape with overlapping ends and pronounced tight junctions (TJ). (**D**–**F**) Astrocytes are bigger than RbE4 cells with less mitochondria (M), but with many caveolae (C), clathrine-coated vesicles (V) and bundled filaments (F). (N—nucleus; G—Golgi apparatus; ER—endoplasmatic reticulum). Magnification (**A**)—3000×; (**B**,**D**)—6000×; (**C**,**E**)—10,000×; (**F**)—12,000×.

**Figure 3:**
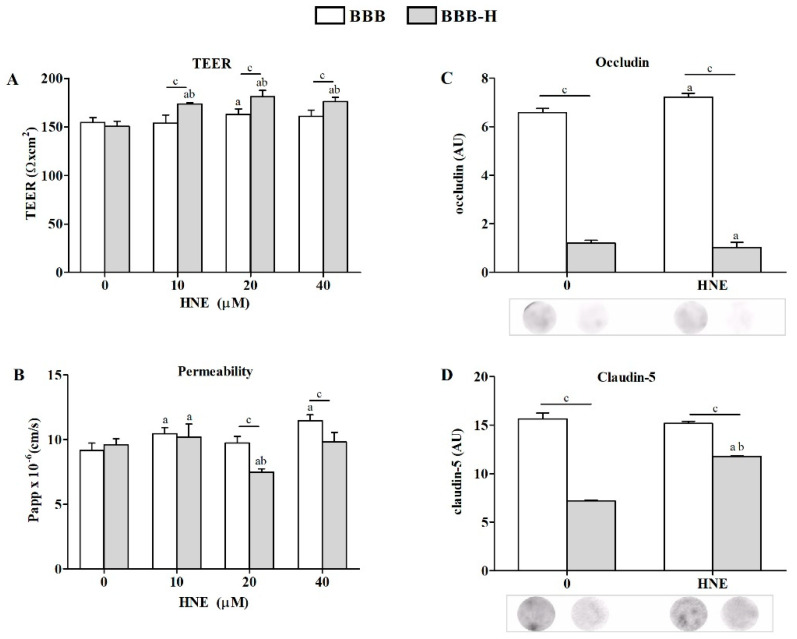
Markers of BBB integrity and permeability measured on BBB and BBB-H model. (**A**) TEER values of both models under HNE treatment (0, 10, 20, 40 µM). (**B**) Permeability of sodium fluorescein for both BBB models under HNE treatment (0, 10, 20, 40 µM). (**C**) Occludin and (**D**) claudin-5 changes in RbE4 cells of both BBB models at control conditions (0) and 20 µM HNE treatment, assessed by dot-blot. Signal intensities were analyzed with NIH ImageJ and represented as arbitrary units (AU). All results are represented as mean ± SD, *n* = 12 samples for each group from 4 independent experiments. a—statistically significant in respect to control (0) on pure collagen, *p* < 0.05; b—statistically significant in respect to control (0) on modified collagen, *p* < 0.01; c—statistically significant between groups of same treatment, *p* < 0.01.

Determining the oxidative status of RbE4 cells on native and HNE-modified collagen was a prerequisite for the development of the following two BBB models: ‘physiological’ later referred to as BBB, and ‘pathological’ referred to as BBB-H. The presence of both cell types (astrocytes and RbE4) on each side of the culture insert membrane was confirmed by electron microscopy for the BBB model (Figure 2). Both BBB models were exposed to HNE in three different concentrations (10, 20, 40 µM), and afterward, TEER and permeability to sodium fluorescein were determined as basic parameters of BBB model integrity (Figure 3A,B). While all HNE concentrations caused an increase in TEER on the BBB-H model (*p* < 0.001) compared to untreated controls of both models, only 20 µM HNE increased TEER in the BBB model. Further, HNE caused a slight but significant increase in the permeability of the BBB model at 10 µM and 40 µM concentrations (*p* < 0.001), without significant effect at 20 µM. On the other hand, 20 µM HNE caused a decrease in the permeability of the BBB-H model (*p* < 0.001). Therefore, subsequent experiments were performed with a 20 µM HNE concentration. First of all, the presence of TJ proteins, occludin and claudin-5, was assessed (Figure 3C,D). In the BBB model, HNE induced a slight increase in occludin (*p* < 0.01) without any detectable effect on claudin-5 levels. Interestingly, in the BBB-H model, expression of both of these proteins was extremely decreased (*p* < 0.001). Although claudin-5 expression was increased after HNE treatment in the BBB-H model, it was still lower compared to the BBB model.

### 2.3. HNE Effects on Lipid Peroxidation and Antioxidant Activity of Endothelial Cells and Astrocytes of Both BBB Models

**Figure 4:**
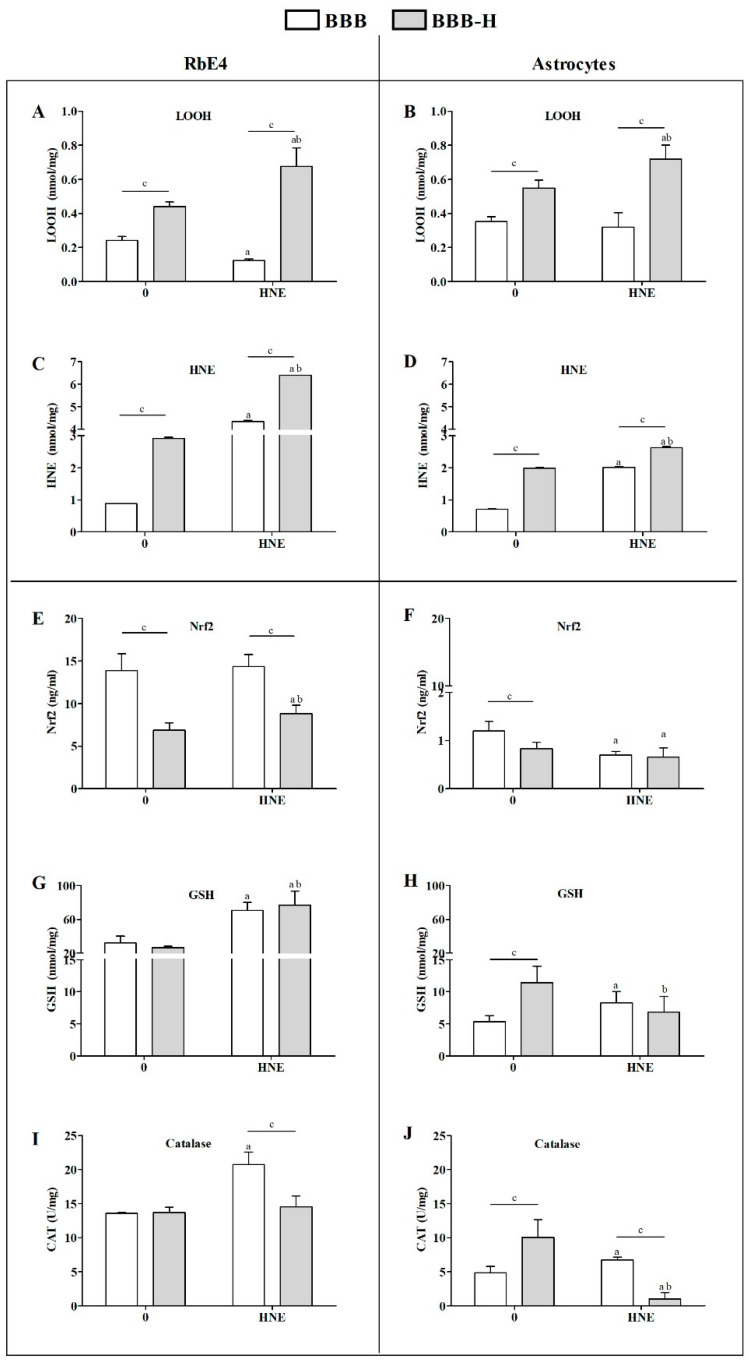
Oxidative and antioxidative status of RbE4 cells and astrocytes of BBB and BBB-H model at control conditions (0) and 20 µM HNE treatment. Measurements of LOOH and HNE-protein conjugates levels were performed to assess oxidative status of RbE4 cells (**A**,**C**) and astrocytes (**B**,**D**) of both BBB models. Nrf2 and GSH levels with CAT activity were measured as main representatives of antioxidative status of RbE4 cells (**E**,**G**,**I**) and astrocytes (**F**,**H**,**J**) of both BBB models. All results are represented as mean ± SD, *n* = 12 samples for each group from 3 independent experiments. a—statistically significant in respect to control (0) on pure collagen, *p* < 0.05; b—statistically significant in respect to control (0) on modified collagen, *p* < 0.01; c—statistically significant between groups of same treatment, *p* < 0.01.

RbE4 and astrocytes in BBB and BBB-H models responded differently to HNE treatment. RbE4 cells in the BBB-H model showed higher concentrations of LOOH (*p* < 0.01) and of HNE-conjugates (*p* < 0.001) but lower Nrf2 expression (*p* < 0.001) than in the BBB model (Figure 4A,C,E). HNE treatment increased LOOH concentrations and HNE-conjugates in BBB-H RbE4 cells (*p* < 0.001). Even though LOOH decreased after HNE treatment in BBB RbE4 cells (*p* < 0.05), amounts of HNE-conjugates increased about four times (*p* < 0.001). Interestingly, GSH levels increased in Rbe4 cells of both models after HNE treatment, while CAT activity increased only in the BBB model after HNE treatment.

HNE affected astrocytes significantly in both models, BBB and BBB-H, even though they were not directly exposed to HNE (Figure 4). Thus, BBB-H astrocytes had higher LOOH concentrations and HNE-conjugates than those of the BBB model (*p* < 0.001) (Figure 4B,D). Although the addition of 20 µM HNE to the RbE4 side of the barrier did not change LOOH concentrations, it did double the number of HNE-conjugates in BBB astrocytes. On the other hand, in BBB-H astrocytes, HNE increased LOOH (*p* < 0.01) together with a slight but significant increase in HNE-conjugates (*p* < 0.05) (Figure 4B,D). The astrocytes of the two models differed in antioxidant status measured by Nrf2 expression, GSH content and CAT activity (Figure 4F,H,J). While BBB astrocytes had higher Nrf2 expression (*p* < 0.01), BBB-H astrocytes showed higher GSH levels and CAT activity (*p* < 0.01). In addition, HNE decreased Nrf2 expression in both models (*p* < 0.001) and had opposite effects on GSH content and CAT activity in our models (Figure 4G–J)—an increase in the BBB model (*p* < 0.01) and a decrease in the BBB-H model (*p* < 0.01).

### 2.4. HNE Affects Lipid Profile of Endothelial Cells and Astrocytes of Both BBB Models

**Figure 5:**
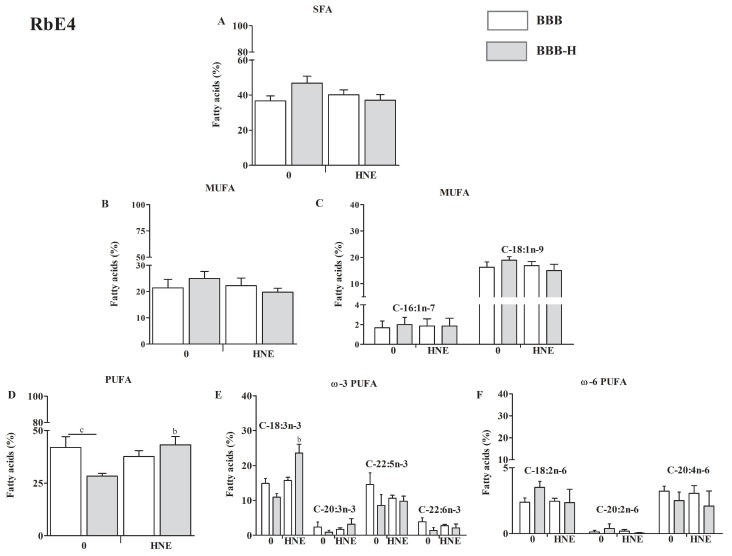
Fatty acid composition of RbE4 cells of BBB and BBB-H model at control conditions (0) and 20 µM HNE treatment based on the degree of saturation: saturated fatty acids (SFA) composition (**A**); monounsaturated fatty acids (MUFA) composition with major representatives (**B**,**C**); polyunsaturated fatty acids (PUFA) composition with major ω3 and ω6 representatives (**D**–**F**). All results are represented as mean ± SEM, *n* = 4 samples for each group from 2 independent experiments. b—statistically significant in respect to control (0) on modified collagen, *p* < 0.01; c—statistically significant between groups of same treatment, *p* < 0.01.

**Figure 6:**
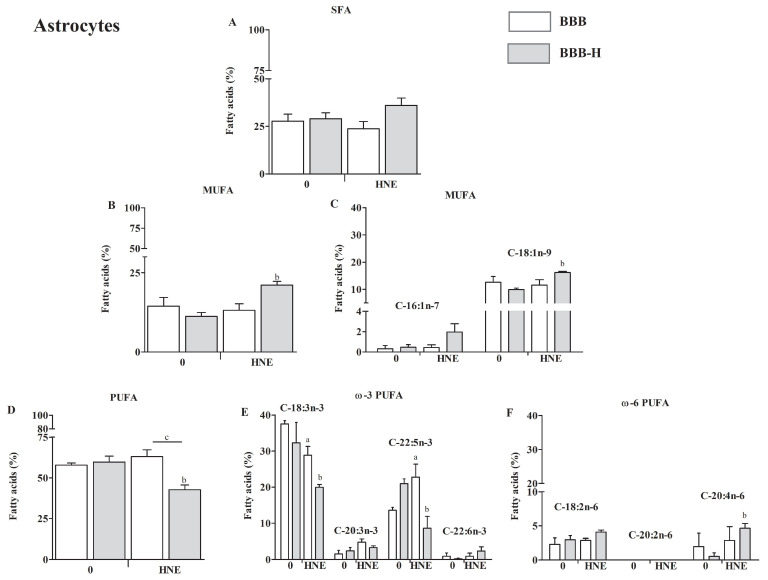
Fatty acid composition of astrocytes of BBB and BBB-H model at control conditions (0) and 20 µM HNE treatment based on the degree of saturation: saturated fatty acids (SFA) composition (**A**); monounsaturated fatty acids (MUFA) composition with major representatives (**B**,**C**); polyunsaturated fatty acids (PUFA) composition with major ω3 and ω6 representatives (**D**–**F**). All results are represented as mean ± SEM, *n* = 4 samples for each group from 2 independent experiments. a—statistically significant in respect to control (0) on pure collagen, *p* < 0.05; b—statistically significant in respect to control (0) on modified collagen, *p* < 0.01; c—statistically significant between groups of same treatment, *p* < 0.01.

Fatty acid composition is presented as a percentage of total lipid content. The main difference in fatty acid composition of RbE4 cells and astrocytes occurred in the degree of saturation. RbE4 cells (Figure 5A,B,D) had lower PUFA content and higher monounsaturated fatty acids (MUFA) and saturated fatty acids (SFA) content than astrocytes (Figure 6A,B,D). 

**Figure A1:**
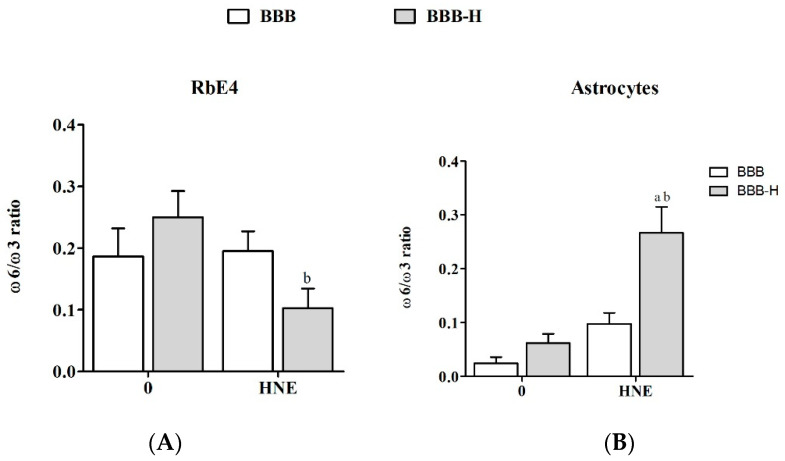
Ratio of ω6 to ω3 fatty acids of RbE4 cells (**A**) and astrocytes (**B**) of BBB and BBB-H model at control conditions (0) and 20 µM HNE treatment. All results are represented as mean ± SEM, *n* = 4 samples for each group from 2 independent experiments. a—statistically significant in respect to control (0) on pure collagen, *p* < 0.05; b—statistically significant in respect to control (0) on modified collagen, *p* < 0.01.

In RbE4 cells, neither SFA nor MUFA did not change significantly regardless of model or HNE treatment (Figure 5A,B). RbE4 cells had different PUFA levels in the two models, BBB and BBB-H, which was the cumulative effect of slight changes in individual PUFAs (*p* < 0.05, Figure 5D–F). HNE treatment increased PUFA levels in the BBB-H model (but did not cause changes in the BBB model), which was a reflection of a significant increase in C-18:3n-3 (ALA, α-linolenic acid, *p* < 0.05 Figure 5D,E). This increase resulted in a decreased ω6 to ω3 ratio in RbE4 cells of the BBB-H model treated with HNE (Figure A1A).

The analysis of SFA content in astrocytes showed no statistical difference regardless of the model analyzed or HNE treatment (Figure 6A). Next, MUFA were significantly increased in the BBB-H model treated with HNE (Figure 6B), which occurred due to elevation in C18:1n-9 (OA, oleic acid, *p* < 0.05, Figure 6C). Further, PUFA did not change in the BBB model, regardless of HNE treatment, and in the BBB-H model (control) compared to the BBB model. A significant decrease in PUFA was observed in the BBB-H model treated with HNE, compared to the BBB-H control and to the BBB model treated with HNE (*p* < 0.05, Figure 6D). Still, these differences show a different pattern for individual PUFA. There is a significant decrease in C-18:3n-3 (ALA, α-linolenic acid) and an increase in C-22:5n-5 (DPA, docosapentaenoic acid) PUFA of BBB model treated with HNE, which combined give no difference in overall PUFA compared to control (BBB model) (Figure 6E). The decrease in PUFA observed in the BBB-H model treated with HNE is a result of a decrease in C-18:3n-3 (*p* < 0.01) and C-22:5n-5 (*p* < 0.01) PUFA. At the same time, an increase in C-20:4n-6 (AA, arachidonic acid, *p* < 0.05) PUFA occurred (Figure 6F). These changes can also be seen in ω6 to ω3 ratio, which increased significantly in astrocytes of the BBB-H model treated with HNE (Figure A1B).

## 3. Discussion

HNE is a pluripotent factor, which can influence cell processes in a wide range of concentrations being present either endogenously or exogenously [21]. Therefore, it is not surprising that HNE was found in the BBB of baboons’ brains under sepsis [22], but also in blood vessels and the stroma of glioma [16,17] indicating a possible role in BBB regulation under stress conditions. Additionally, in an in vitro BBB model, HNE was described as a harmful molecule that induces BBB permeability [23]. In such a severe endothelial barrier dysfunction states, HNE was shown to directly form adducts with adherent and tight junction proteins and integrins [24]. Therefore, our aim was to investigate the mechanism by which HNE changes BBB function while being present either in the bloodstream or in the ECM, with an accent on the lipid composition of endothelial cells and astrocytes. For this purpose, an artificial BBB was established as a model from both endothelial cells and astrocytes, reflecting the in vivo BBB.

In order to establish a simple but effective BBB model, first the growth characteristics of RbE4 cells on collagen and HNE-modified collagen in combination with HNE treatment were studied. Measurements of ROS and LOOH indicated that 10 µM HNE caused the greatest differences between the two surfaces. This HNE concentration is described in the literature as the highest physiological concentration used in vitro that beneficially affects the cellular signaling pathways or induces an adaptive cell response to stress [21]. Next, we moved to BBB models, where TEER and permeability showed that indeed the BBB model was functional and HNE treatment on the RbE4 side of the BBB-H caused an increase in TEER and a decrease in the permeability of the BBB. At the same time, occludin and claudin-5 levels remained low in the BBB-H model compared to the BBB model, even though a slight increase in claudin-5 expression in the BBB-H model occurred after HNE treatment. These two proteins are integral TJ proteins, whose levels impact BBB permeability [25]. However, occludin loss can be surmounted by other TJ proteins, thus its level is not crucial for BBB function [26]. In contrast to occludin, claudin-5 is one of the key molecules that contribute to TJ in a way that its decrease is associated with increased BBB permeability, while its increase results in the strengthening of barrier properties [27]. Taking these data into consideration, our results indicate that low levels of ECM stress cause a decrease in both, occludin and claudin-5, but not enough to significantly disrupt the barrier properties. The decrease in PUFA levels, which was followed by an increase in SFA and MUFA in RbE4 cells of the BBB-H model, possibly reflected the rigidity of the membrane and surpassed this decrease in occludin and claudin, thus maintaining the BBB properties. On the other hand, HNE treatment has low or no influence on these molecules in native BBB, while in BBB-H it slightly, but significantly decreased occludin and significantly increased claudin-5. HNE is a known activator of EGFR (endothelial growth factor receptor) [28], and active EGFR can suppress occludin transcription and translation by downstream activation of p38 MAPK/NF-κB [29]; therefore, this pathway should be further investigated. Further, HNE increased the claudin-5 level in the BBB-H model, which is in concordance with the aforementioned barrier strengthening. Still, this increase did not reach the control levels of the BBB model, but PUFA, MUFA and SFA values returned to control levels in RBE4 cells together with lipid changes in astrocytes that included a decrease in PUFA followed by an increase in MUFA. The importance of lipid composition in the regulation of BBB permeability was emphasized in the BBB parallel artificial membrane permeability assay, which showed that lipid composition markedly influences passive permeability [30]. Besides the obvious effect on BBB integrity and permeability, HNE modification of collagen caused profound changes in the oxidative status of RbE4 cells and astrocytes and also in their PUFA composition. BBB-H RbE4 cells had higher LOOH and HNE-protein conjugate levels as expected, but this pattern was also observed for astrocytes, which were not directly exposed to HNE-modified collagen. The addition of exogenous HNE increased these differences. Even more, interesting results occurred in the antioxidative status of both cell types. BBB astrocytes had almost ten times lower Nrf2 levels than RbE4 cells while in the BBB-H model this difference was not so profound. These results could be due to the function of endothelial cells, which are the first line of defense at the blood–brain barrier, resulting in higher GSH and CAT levels compared to the brain [31]. HNE did not cause changes in Nrf2 levels of BBB RbE4 cells, even though studies showed an increase in Nrf2 levels in HUVEC upon HNE exposure [32]. However, increased levels of GSH and CAT activity after HNE treatment, suggest that Nrf2 could have been activated without an increase in its overall level, showing sufficient basal levels to compensate for low levels of stress. Surprisingly, BBB-H RbE4 cells had two times lower Nrf2 levels than those of the BBB model. Since Nrf2 regulation also depends on adhesion molecules [33], and HNE caused a decrease in occludin and claudin-5, it is possible that HNE diffused from HNE-modified collagen and altered junctional proteins. The study of Xu et al**.** favors this assumption, as they showed that HNE bound to fibronectin altered the expression of surface integrins and adhesion molecules [34]. Fast recovery of GSH levels in astrocytes after HNE treatment was also shown in optical head nerve astrocytes [35]. These authors showed a relative increase in Nrf2 mRNA up to 3 h after HNE exposure, but this increase was not translated to the protein level. Astrocytes are sensing changes in their proximity and actively responding, thus protecting the CNS from oxidative stress through an increase in enzymes of GSH synthesis, resulting in higher GSH levels that can be released in the ECM for neuron protection [36]. This could easily explain the observed changes in GSH and CAT activities in our models. Besides changing the antioxidative profile, astrocytes can react by secreting different cytokines, chemokines and neurotrophic factors, but also, which is rarely mentioned, through lipid composition changes [37]. Additionally, astrocytes readily release PUFA, which are taken by neurons or endothelial cells due to their lack of enzymes needed for desaturation and elongation in creating new PUFA in these cells [38,39,40]. This fine cooperation between endothelial cells and astrocytes also became apparent in our study. PUFA, especially ω-3 PUFA, decreased in RbE4 cells on HNE-modified collagen. Exogenous HNE reversed this effect by inducing ALA. At the same time, in BBB-H astrocytes, exogenous HNE reduced PUFA levels, again dominantly ω-3 (ALA, DPA), and increased MUFA (OA). Since ALA is an essential fatty acid and thereby cannot be synthesized de novo [41], it can be assumed that RbE4 cells incorporated ALA released by astrocytes. Decreased ALA levels without an increase in ALA metabolites in astrocytes support this assumption. Unfortunately, little is known on HNEs impact on PUFA composition or direct interactions with lipids since the majority of studies are dedicated to revealing HNE-modified proteins and their implications in cell signaling. For it is known, PUFA composition is altered in brain tumors in favor of AA and decrease in DHA [42]. With deregulated fatty acid uptake and lipid metabolism found in malignant glioma, and an increase in the AA:DHA ratio, one can assume that tumorigenicity is closely associated with DHA loss [43]. Therefore, studies of PUFA metabolism in different diseases are needed to elucidate the relevance of the observed changes.

All the above mentioned emphasizes the importance of the role of HNE not only in the development and onset of CNS diseases, but more importantly, its ability to restrict drug flux across the BBB by directly causing significant changes in the lipid composition of endothelial cells and indirectly in astrocytes. This pluripotent reactive aldehyde, which has the ability to modify proteins and subsequently signaling pathways and cellular processes, demonstrated in this study its important ability to influence the transport properties of the BBB through changes in lipid composition. This study brings possible explanations for the treatment failure of CNS diseases and opens new opportunities for further clarification of this major problem.

## 4. Materials and Methods

### 4.1. Cell Culture Conditions and Treatments

Primary culture of astrocytes between passages 2 and 4 (kindly provided by prof. N. Žarković) were used for all experiments and cultivated in Dulbecco’s modified Eagle’s medium (DMEM, Sigma Aldrich, St. Louis, MO, USA) with 10% Fetal calf serum (FCS, Sigma Aldrich, St. Louis, MO USA), 2 mM glutamine (Sigma Aldrich, St. Louis, MO, USA), 100 U/mL penicillin (Pliva, Zagreb, Croatia), 100 µg/mL streptomycin (Pliva, Zagreb, Croatia). Before each experiment astrocyte culture was verified for glial fibrillary acidic protein (GFAP, Dako, Glostrup, Denmark). After reaching confluence, astrocyte conditioned medium (ACM) was collected for further experiments. In each experiment, astrocytes (seeded at 50,000 cells/cm^2^) were grown for 10 days during which the medium was changed every other day.

Cloned immortalized rat brain endothelial cells (RbE4) (a generous gift of prof. *p*. Couraud, Paris, France) were cultivated in Minimum essential medium/Ham’s F10 1:1 (MEM/F10, Sigma Aldrich, St. Louis, MO, USA), 10% FCS, 2 mM glutamine, 1 ng bFGF (Peprotech, Vienna, Austria), 300 µg/mL geneticin (SCBT, Heidelberg, Germany) and grown on rat tail collagen-coated Petri dishes until confluence. For all experiments, RbE4 cells (180,000 cells/cm^2^) were seeded in MEM/F10 and left for 24 h to attach. Afterwards, cells were grown in a mixture of MEM/F10 and ACM (1:1) during seven days. The mixture of media was changed every other day. Cells were grown under two conditions–on collagen coated dishes, or dishes coated with collagen treated with 10 µM HNE (kindly provided by prof. K. Uchida).

HNE treatments were performed in a culture media without FCS for each cell culture. After one-hour incubation, FCS was added to the treatments (to final concentration 10% (*v*/*v*)) and left for 24 h until analysis or sample collection.

### 4.2. Cell Viability and Proliferation Assays

For viability and proliferation assays cells were seeded in 96-well plates and were treated with HNE in a range of concentrations (1, 5, 10, 20, 40, 50, 75, 100 µM). Viability was measured 24 h after treatment by MTT assay (EZ4U, Biomedica GmBh, Vienna, Austria) according to manufacturer’s instructions [44]. Proliferation of the cells was determined as the incorporation rate of radioactive [^3^H]thymidine into DNA of the cells. One hour after HNE treatment and addition of FCS, radioactive [^3^H]thymidine ([6-^3^H]thymidine; Amersham Biosciences, Amersham, UK) was added at 0.25 µCi to each well and incubated for the next 24 h [^3^H]thymidine incorporation was measured as described elsewhere [45].

### 4.3. Sample Preparation for Protein and Lipid Based Assays

For all analyses cells were trypsinized and cell pellets were stored at −80 °C until protein or lipid isolation. Cell pellets were resuspended in phosphate buffer solution (PBS) and proteins were isolated by 4 freeze and thaw cycles followed by centrifugation at 13,500× *g* for 10 min. Prior to all analyses, protein concentration in each sample was measured by the Bradford method [46]. Lipids were isolated also from cell pellets resuspended in PBS according to modified Folch method [47]. Briefly, after addition of chloroform (5 mL) samples were mixed roughly to ensure optimal mixing of aqueous and hydrophobic phases. Prior to centrifugation, aqueous solution of magnesium chloride (1.5 mL; 0.034%, *w*/*v*) was added and samples were vortexed. Upper aqueous phase was removed and 2 M solution of potassium chloride in methanol (2.5 mL; 4:1, *v*/*v*) was added. After vortexing and centrifugation, aqueous phase was removed once again, and chloroform/methanol solution (2.5 mL; 2:1, *v*/*v*) was added followed by vortexing and centrifugation. Lower hydrophobic phase was then transferred to new glass tubes and evaporated in the nitrogen gas stream. Dry residues were stored at −80 °C until further analysis.

### 4.4. ROS and LOOH Measurements

Cellular ROS production was measured in RbE4 cells by a method dependent on intracellular deacylation and oxidation of 2,7-dichlorodihydrofluorescein diacetate (DCFH-DA, Fluka, Charlotte, NC, USA) to the fluorescent compound 2,7-dichlorofluorescein (DCF). Briefly, after entering cells, cellular esterases remove the acetyl group from DCFH-DA, thereby forming the non-fluorescent DCFH, which is then oxidized by ROS to fluorescent DCF. The method measures the total cellular ROS that are capable to oxidize DCFH. After being cultivated for seven days, cells were washed in Hank’s balanced salt solution (HBSS) and incubated with 100 µM DCFH-DA for 30 min. Thereafter, DCFH-DA solution was removed from the cells and they were washed with HBSS and treated with HNE (10, 20, 40 µM). ROS was measured during 24 h (0, 1, 3, 24 h) on spectrofluorimeter Varian Cary Eclipse (Varian NMR Instruments, Palo Alto, CA, USA) (λ_ex_ = 500 nm, λ_em_ = 529 nm).

Lipid hydroperoxides (LOOH) were measured in RbE4 cells grown as monolayer as well as in RbE4 cells and astrocytes of both BBB models. Cells were collected 24 h after HNE treatment (10, 20, 40 µM) by trypsinization and lipids were isolated as described in Section 2.3. LOOH were determined by ferric thiocyanate spectrophotometric method [48]. All measurements were performed by the UV/Vis spectrophotometer Varian Cary 4000 (Varian NMR Instruments, Palo Alto, CA, USA).

### 4.5. BBB Integrity Evaluation

**Figure A2:**
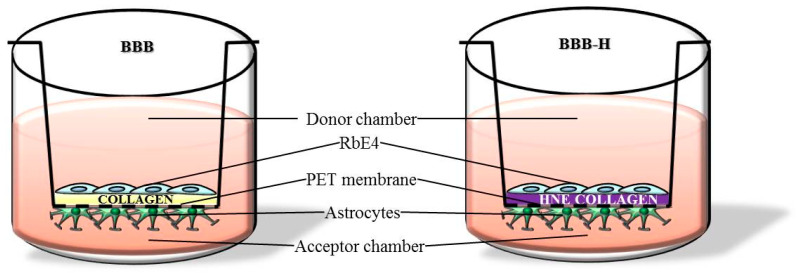
Schematic representation of BBB and BBB-H models.

BBB models were established on cell culture inserts (12 well P.E.T. inserts, 0.45 µm pore size, BD Falcon, Bedford, MA, USA) as described [23]. Briefly, culture inserts were coated with collagen, or with collagen treated with 10 µM HNE a day before astrocyte seeding. Astrocytes were seeded on the bottom side of the insert, and three days later, RbE4 cells were seeded on the upper side of the insert. Model designated as BBB was made on collagen-coated inserts and represents ‘physiological’ BBB model, while model designated as BBB-H was made on inserts coated with collagen treated with 10 µM HNE and represents ‘pathological’ BBB model (schematic representation—Figure A2).

As a test of the BBB permeability, transport of the paracellular marker sodium fluorescein (Sigma Aldrich, St. Louis, MO, USA) across the layer was determined. Sodium fluorescein (50 µM) prepared in HBSS was added to the donor chamber and colorless HBSS was added to the acceptor chamber. After 1 h amount of sodium fluorescein in both chambers was determined using spectrofluorimeter Varian Cary Eclipse (λ_ex_ = 460 nm, λ_em_ = 515 nm). The passage of sodium fluorescein was expressed as apparent permeability (Papp) in cm/s according to the following equation [49]: *P_app_* = (*V_b_ × dc*)/(*dt × A × C*_0_), where *V_b_* is a volume of acceptor side (mL); dc is the change of the sodium fluorescein in acceptor chamber (µM); dt is the time change (s); A is the surface of the membrane (cm^2^); *C_0_* is the initial concentration of sodium fluorescein in the donor chamber (50 µM).

Trans-endothelial electrical resistance (TEER) reflecting the flux of mainly sodium ions through cell layers in culture conditions was measured by Epithelial-volt-ohm meter (World Precision Instruments, Sarasota, FL, USA) and expressed as Ω cm^2^. TEER of coated, but cell-free inserts were subtracted from measured TEER values of the models.

### 4.6. Electron Microscopy

Cells grown on the inserts were fixed with solution of 2% glutaraldehyde and 2% paraformaldehyde in cacodylate buffer (pH7.5) for 30 min. After washing with cacodylate buffer, cells were postfixed in 1% osmium tetroxide for 60 min. Following washing with distilled water, cells on the insert were dehydrated in graded acetone and block-stained with 2% uranyl acetate in 50% ethanol for 1 h. The membrane of the culture insert, with the cells on the two sides, was removed from its support and embedded in the epoxy resin. Ultrathin sections were cut perpendicularly for the membrane using a Leica ultracut UCT ultramicrotome (Leica Microsystems, Wetzlar, Germany) and examined using a transmission electron microscope (Morgagni 268D, Philips, Amsterdam, Netherlands).

### 4.7. Total Glutathione and Catalase Activity Assays

Total GSH was measured based on a kinetic reaction in which GSH causes a continuous reduction of 5,5-dithiobis (2-nitrobenzoic acid) to yellow 5-thio-2-nitrobenzoic acid [50]. Formed GSSG is recycled by glutathione reductase and NADPH, improving the sensitivity of total glutathione detection. The yellow product is then measured spectrophotometrically at 405 nm. The total glutathione levels were determined from a standard curve of reduced glutathione and expressed as nanomoles of GSH per milligram of protein.

Activity of catalase (CAT) was measured in 40 µL of samples with protein content adjusted to 30 µg/well. Assay is based on the disappearance of hydrogen peroxide in the presence of CAT at room temperature [51]. Ammonium molybdate (32.4 mM) was used as a stop solution, and color development was measured spectrophotometrically in a plate reader at 405 nm.

### 4.8. Dot Blot Analysis

The dot-blot analyses were performed in cell lysates with adjusted protein content to 100 μg/mL. Briefly, 100 μL of each sample was loaded on a nitrocellulose membrane (Bio-Rad Laboratories, Hercules, CA, USA) and washed twice with PBS before blocking the membrane for 1 h at room temperature (RT). Blocking solution for HNE determination was 2% (*w*/*v*) nonfat dry milk (Bio-Rad Laboratories, Hercules, CA, USA) in PBS, while for Nrf2, occludin and claudin-5 determination solution of 1% nonfat dry milk, 1% BSA, 0,05% Tween 20 (*w*/*w*/*v*) in Tris buffer solution (TBS) was used. The membrane was then incubated for 2 h at RT with non-commercial genuine monoclonal mouse anti-HNE-histidine antibody (1:100; clone HNE 1 g4, a generous gift of prof. G. Waeg) [52] or 1 h at RT for rabbit polyclonal anti-Nrf2 (1:50; sc-722; Santa Cruz Biotechnology, Heidelberg, Germany), rabbit polyclonal anti-occludin (1:50; sc-5562; Santa Cruz Biotechnology, Heidelberg, Germany), rabbit polyclonal anti-claudin-5 (1:500; ab53765; Abcam, Cambridge, UK). After blocking of endogenous peroxidases, the membrane was incubated with a secondary antibody (1:25; EnVision K4001 for anti-HNE or 1:10; EnVision Flex K8000 for anti-occludin and anti-claudine-5; Dako North America, Carpinteria, CA, USA) for 1 h at RT. Immune complexes were visualized using 3,3′-diaminobenzidine (DAB; Dako North America, Carpinteria, CA, USA) and scanned for quantification of signals. Signals were analyzed in NIH ImageJ (U.S. National Institutes of Health, Bethesda, MD, USA, a Java-based image processing program.

Amounts of HNE-protein conjugates were recalculated from the standard curve prepared with HNE-BSA standards (0; 0.1; 0.5; 1; 2.5; 5; 10; 20; 30; 40; 50; 100 nmol/mg) and expressed as nmol of HNE-protein adducts/mg of proteins. Amounts of Nrf2 were recalculated from the standard curve prepared with Nrf2 protein standards (0; 1.96; 3.91; 7.81; 15.63; 31.25; 62.5; 125; 250 ng/mL) and expressed as ng/mL.

### 4.9. Lipid Analysis

The lipid extract was treated with 0.5 M solutions of potassium hydroxide in methanol [53] for 10 min at RT and the corresponding fatty acid methyl esters (FAMEs) were formed, extracted with n-hexane and examined by gas chromatography (GC). GC analyses of total fatty acids were performed by using Varian 450-GC equipped with a flame ionization detector (Varian BV, Middelburg, Netherlands). A Stabilwax column (crossbond carbowax polyethylene glycol, 60 m × 0.25 mm, Restek, Bellefonte, PA, USA) was used as a stationary phase with helium as a carrier gas. The heating was carried out at a temperature of 150 °C for 1 min followed by an increase of 5 °C/min up to 250 °C. The methyl esters were identified by comparison with retention times of standard mixtures commercially available (Marine oil FAME mix, Restek, Bellefonte, PA, USA). Individual fatty acids were expressed as relative percentages of FAME obtained from total lipid extract. The relative amount of each fatty acid (percentage of FAME) was quantified by integrating the area under the peak and dividing the results by the total area for all fatty acids.

### 4.10. Statistical Analyses

All data are reported as mean ± SD. Significant differences between means were assessed depending on experiment settings by one-way ANOVA or two-way ANOVA. As *post hoc* tests, Dunnett test was performed for comparing means to control group and Bonferroni test for multiple comparisons of means between groups. A *p* value less than 0.05 was considered statistically significant.

## Data Availability

The data presented in this study are available in article.
